# Impacts of Sulfur on Glucosinolate Metabolism: From *Arabidopsis* to Wild Brassicales

**DOI:** 10.3390/plants14142129

**Published:** 2025-07-10

**Authors:** Villayat Ali, Takeru Yoshimoto, Dhiraj Vyas, Akiko Maruyama-Nakashita

**Affiliations:** 1 Plant Science and Agrotechnology Division, Indian Institute of Integrative Medicine, Jammu 180001, India; makpie90@gmail.com (V.A.); dvyas@iiim.res.in (D.V.); 2 Department of Bioscience and Biotechnology, Faculty of Agriculture, Kyushu University, 744 Motooka, Nishi-ku, Fukuoka 819-0395, Japan; yoshimoto.takeru.961@s.kyushu-u.ac.jp; 3 Academy of Scientific and Innovative Research, Ghaziabad 201002, India

**Keywords:** Brassicales, glucosinolates, sulfur, metabolism, sulfur limitation, glucosinolate hydrolysis products, *Arabidopsis thaliana*, wild perennial, *Lepidium latifolium*, environmental factors

## Abstract

Glucosinolates (GSLs), sulfur-containing secondary metabolites produced by cruciferous plants, act as sulfur (S) stores, repel pests, and exhibit cancer-preventive properties in humans. Based on *Arabidopsis* studies, this study outlines the regulatory mechanisms of GSL biosynthesis and metabolism in response to environmental S content. Additionally, we review the case of wild perennial Brassicales, with a focus on *Lepidium latifolium*. These wild perennial species respond differently to S availability by modulating their GSL profiles to enhance insect interactions or allocate resources for survival. The rich diversity of GSLs among wild perennial Brassicales highlights the potential for uncovering the ecological and physiological roles of GSLs and their catabolites in plants, as well as their nutraceutical benefits for human health.

## 1. Introduction

**Figure 1:**
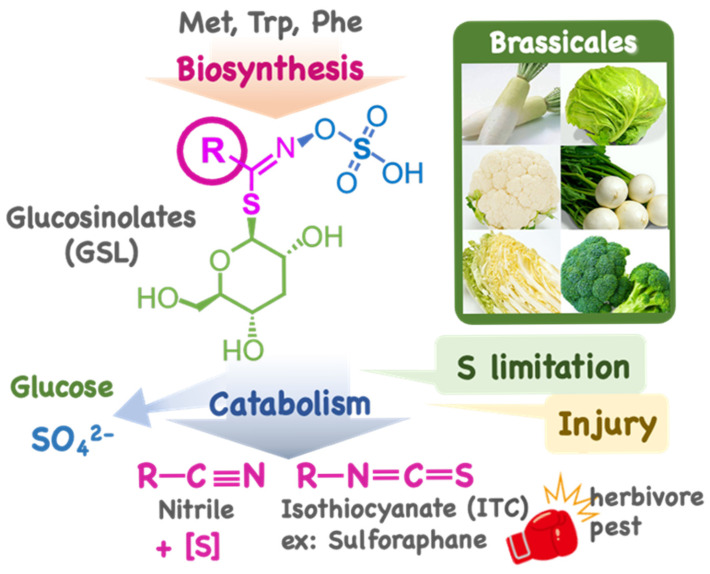
Structure and metabolism of glucosinolates (GSLs). The structure of GSLs consists of a sulfate group (blue), a glucose moiety (green), and an S-C-N bonding structure attached to a side chain (pink). Glucosinolates (GSLs) have the basic structure of a sulfated oxime group bound to thioglucose, with a side chain (R) derived from an amino acid. They are biosynthesized from amino acids such as methionine (Met), tryptophan (Trp), and phenylalanine (Phe); those derived from Met are called aliphatic GSLs (mGSLs), while those derived from Trp are called indolic GSLs (iGSLs). Benzenic GSLs are derived from Phe and/or Tyr. GSLs are catabolized into isothiocyanates (ITCs) or nitriles in response to tissue damage and a lack of sulfur (S). The released glucose and sulfate are recycled in primary metabolism. In the metabolic process to nitriles, one more S is released.

Plant-specific metabolites contribute to unique environmental adaptations and survival strategies and play key roles in organismal diversity. Glucosinolates (GSLs) are a type of plant-specific sulfur (S)-containing metabolite produced by members of the Brassicaceae family, known as cruciferous plants (Figure 1). The model plant *Arabidopsis thaliana*, as well as cabbage, Chinese cabbage, radish, turnip, and broccoli, etc., belong to the Brassicaceae family; therefore, GSLs are among the most extensively studied plant-specific metabolites [1,2,3,4]. The characteristic odor and pungency of *Brassica* crops are caused by isothiocyanates (ITCs), which are degradation products of GSLs [5]. GSLs are hydrolyzed by a group of ß-glucosidases called myrosinases. In general, GSLs and myrosinases are stored in separate cells or separated organelles in cells. However, when they mix due to tissue damage, GSL hydrolysis occurs and ITC is produced, the so-called mustard oil bomb [6]. ITC is toxic to a wide range of organisms and functions as a defense mechanism against predators and infection [7,8]. ITCs also function as phytochemicals in humans [9,10,11]. Sulforaphane, a breakdown product of 4-(methylsulfinyl)butyl GSL (glucoraphanin), is well-known and is marketed as a functional food and supplement. In contrast, ITCs such as goitrin, a catabolite of 2-hydroxy-3-butenyl GSL (progoitrin), can be goitrogenic to humans and livestock [4]. Because GSLs accumulate in seeds, low-GSL varieties have been bred for rape seeds, a typical oil crop worldwide [4].

**Figure 2:**
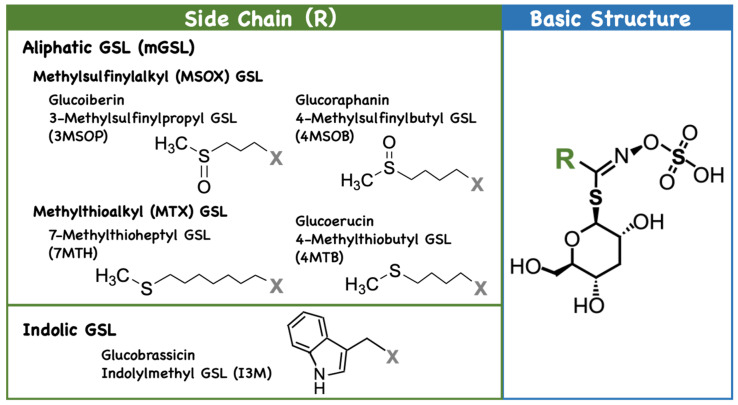
Examples and classification of glucosinolates (GSLs). “X” in the left panel indicates the basic structure depicted in the right panel. “R” in the right panel represents the side chain, with examples illustrated in the left panel.

GSLs consist of a basic backbone of sulfated oxime group attached to thioglucose, with side chains derived from amino acids [1,2,3,4]. They are divided into aliphatic, indolic, and benzenic GSLs that are synthesized from methionine (Met), tryptophan (Trp), and phenylalanine (Phe) or tyrosine (Tyr), respectively [12,13]. In *Arabidopsis*, approximately 40 GSLs have been reported [14], mainly aliphatic GSLs (mGSLs) derived from Met and indolic GSLs (iGSLs) derived from Trp (Figure 2). mGSLs accumulate mostly in the aboveground parts, whereas iGSLs, represented by indol-3-ylmethyl GSL (I3M) and 1- or 4-methoxy I3M (1/4MOI3M), accumulate mostly in the roots [15,16]. mGSLs are further classified into methylsulfinylalkyl (MSOX) GSLs and methylthioalkyl (MTX) GSLs according to their structures (Figure 2) [2,3,4,14]. The former include 3-(methylsulfinyl) propyl GSL (3MSOP), 4-(methylsulfinyl) butyl GSL (4MSOB), 7-(methylsulfinyl) heptyl GSL (7MSOH), and 8-(methylsulfinyl) octyl GSL (8MSOO), while the latter include 4-(methylthio) butyl GSL (4MTB), 7-(methylthio) heptyl GSL (7MTH), and 8-(methylthio) octyl GSL (8MTO). In *Arabidopsis thaliana* cv. Col-0, MSOX is the major mGSL in leaves, whereas MTX is more abundant in seeds [15,16]. The significance of such site-specific GSL distribution is not well-understood but is the result of GSL biosynthesis, catabolism, and transport [17]. As GSL transporters, GSL TRansporter (GTR) 1, GTR2, and GTR 3, which take up GSL into cells [17,18], and Usually Multiple Acids Move In Out Transporter (UMAMIT) 29, UMAMIT30, and UMAMIT31, which export GSLs from cells [19,20], have been identified. They cooperatively work to transport GSLs to seeds, resulting in high concentrations of GSLs [20].

Recently, the GSL functions of storage, particularly S storage, have been highlighted. Various factors influence sulfate availability in the soil; approximately 50 mg SO_4_^2−^ L^−1^ (0.52 mM) is required in hydroponic solutions, with the dry weight (DW) decreasing below 10 mg SO_4_^2−^ L^−1^ (0.10 mM) [21]. The total S content in the soil varies between 50 and 100,000 mg S kg^−1^, with surface soils containing 56–618 mg S kg^−1^ [22]. The supply of S to the agro-environment has continued to decline since the early 2000s due to the enhanced desulfurization efficiency of petroleum fuels reducing the release of S into the environment, the use of fertilizers with lower S contents, and a decrease in the use of S-containing pesticides. Depending on crop species, crop S requirements range from 0.10% to 6% (0.03 and 2 mmol g^−1^) of DW [22]. Brassica crops have particularly high S requirements, with seed ranging from 1.1–1.7%, and GSL levels varying with S supply [21]. In this review, we summarize recent findings on the contribution of GSLs to S nutritional management in plants, starting from the knowledge obtained from the model plant *Arabidopsis*, and then from other wild perennial Brassicales.

## 2. Effect of S Nutrition on GSL Biosynthesis and Metabolism

As inferred from their high accumulation in seeds, GSLs act as storage substances. In Brassica napus plants, GSLs account for 10–30% of the organic S content [4,23]. The GSL content in plants fluctuates according to S availability in the environment and S fertilization [23], with S deficiency (-S) significantly decreasing the amounts of GSLs [24,25]. The contribution of GSLs to S storage is supported by the initial growth retardation in GTR1 and GTR2 disruption lines, which accumulate much less GSLs than wild-type plants [16]. Therefore, GSL biosynthesis and metabolism are presumed to be regulated by S at the molecular level. In addition, the glucose moiety in GSLs and the aliphatic side chain in mGSLs can function as carbon storage sites [26,27].

**Figure 3:**
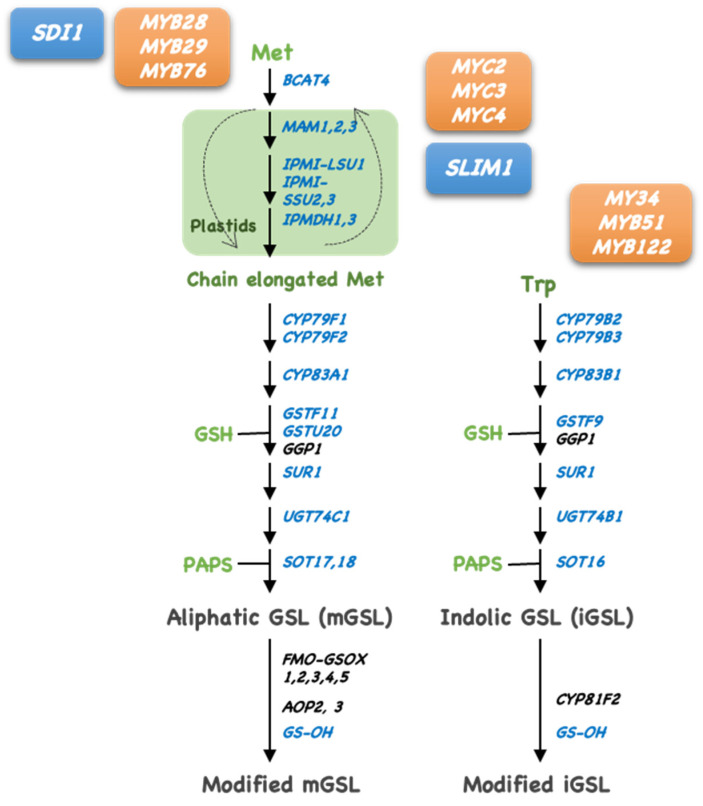
Biosynthetic pathways for glucosinolates (GSLs). Left: aliphatic GSL (mGSL); Right: indolic GSL (iGSL). The enzyme names are shown in italics. Enzyme names for which transcript levels decrease below -S are shown in blue; S-containing metabolites except for GSLs are shown in green; transcription factors that promote GSL biosynthesis are shown on orange backgrounds; transcription factors and regulatory proteins that repress GSL biosynthesis are shown on a blue background.

GSLs are biosynthesized from amino acid precursors through the chain elongation of the precursor amino acid, formation of a central structure, and side chain modifications (Figure 3) [2,3,4]. mGSL biosynthesis involves Met-chain elongation in plastids. Met undergoes deamination by branched-chain amino acid aminotransferases (BCATs) to form 2-oxoacid. The resulting 2-oxoacid is condensed with acetyl-CoA by methylthioalkylmalate synthase (MAM), isomerized by isopropylmalate isomerase (IPMI), and oxidatively decarboxylated by isopropylmalate dehydrogenase (IPMDH) to release 2-oxoacid. This cycle was repeated to elongate aliphatic Met side chains.

After chain elongation, Met is activated to aldoxime and nitrile oxide by cytochrome P450 (CYP79 and CYP83) and conjugated with glutathione (GSH) by GSH-S-transferase (GST). Then, γ-glutamyl peptidase (GGP1) liberates glutamate, and S-alkyl-thiohydroxy acid lyase (SUR1) forms a thiol group. The addition of glucose to this thiol group generates desulfo-GSL, and then sulfotransferase (SOT) adds a sulfate group from 3′-phosphoadenosine 5′-phosphosulfate (PAPS) to desulfo-GSL. GSLs undergo further side-chain modification via a flavin-containing monooxygenase (FMO-GSOX)-catalyzed reaction to form MSOX from MTX.

The gene expression of these GSL biosynthetic enzymes is promoted by the MYB transcription factors MYB28, MYB29, and MYB76 for mGSL biosynthesis, and MYB34, MYB51, and MYB122 for iGSL biosynthesis [3,28,29,30] (Figure 3). MYC2, MYC3, and MYC4 also promote GSL biosynthesis by interacting with these MYBs (Figure 3) [31]. The gene expression of GSL biosynthetic enzymes is significantly reduced by -S [24,25]. Sulfur LIMitation (SLIM)1, which belongs to the plant-specific transcription factors Ethylene-Insensitive3-Like (EIL) family and is identical to EIL3, is a transcriptional regulator responsible for the repression of GSL biosynthesis under –S [24,32]. SLIM1 is a major regulator of the -S response, which represses GSL biosynthesis and stimulates S assimilation and GSL catabolism in response to –S [24,32]. However, the transcript levels of SLIM1 do not highly modulated under -S; the mechanism by which GSL decreases in response to -S had been remained a question [24,32].

## 3. Suppression of GSL Biosynthesis in Response to S Deficiency

Several functionally unknown transcripts increase under -S [30,32,33,34], including those classified to the gene families *Response to Low Sulfur* (*LSU*) and *Sulfur Deficiency Induced* (*SDI*). A reverse genetic analysis of these proteins revealed that SDI repressed mGSL biosynthesis [25]. *SDI1* and *SDI2* expressions were significantly induced by -S. They have a tetratricopeptide repeat (TPR) that facilitates protein–protein interactions, and SDI1 possesses a nuclear localization signal. In the *SDI1* and *SDI2* double-disruption lines (*sdi1sdi2*), the transcript levels of mGSL biosynthetic genes increased, as did the mGSL levels, while they decreased in the over-expression lines of *SDI1* and *SDI2* [25]. The influences of *SDI1* expression were greater than those of *SDI2* expression in both disrupted and over-expressed lines.

The presence of the TPR domain inspired SDI to interact with specific proteins. The interaction between SDI1 and MYB28 has been demonstrated in both yeast and the nuclei of plant cells, as well as in the electromobility shift assay [25]. SDI1 was capable of binding to MYB28 on the mGSL biosynthetic genes promoter without disturbing the DNA binding ability of MYB28 and repressed the transcription-promoting activity of MYB28, resulting in the suppression of mGSL biosynthetic genes, leading to mGSL biosynthesis [25]. Similarly, SDI1 was exclusively responsible for the repression of mGSL biosynthesis under -S [25]. This flexible and reversible system enables plants to regulate mGSL biosynthesis in a fluctuating S environment. However, the molecular mechanisms through which SDI1 represses the activity of MYB28, SDI2 (which is not localized in the nucleus) represses GSL biosynthesis; the -S-induced *SDI* gene expressions [35] and the relation with other transcription factors possibly involved in GSL biosynthesis [36,37,38] require further research.

Interestingly, SDI homologs also exist in plants that do not accumulate mGSL, and their gene expression is induced by -S. SDI should have functions other than the repression of mGSL biosynthesis. Likewise, SDI1 suppresses the accumulation of S-containing seed storage proteins [39]. SDI1 represses the expression of seed storage proteins by forming a complex with MYB28 and MYC2, transcription factors regulate seed storage protein accumulation [31,39,40]. These findings suggest that, under -S, more proteins that are unfavorable for plant survival under -S interact with SDI and repress S-consuming metabolic processes.

## 4. Induction of GSL Catabolism by S Deficiency

**Figure 4:**
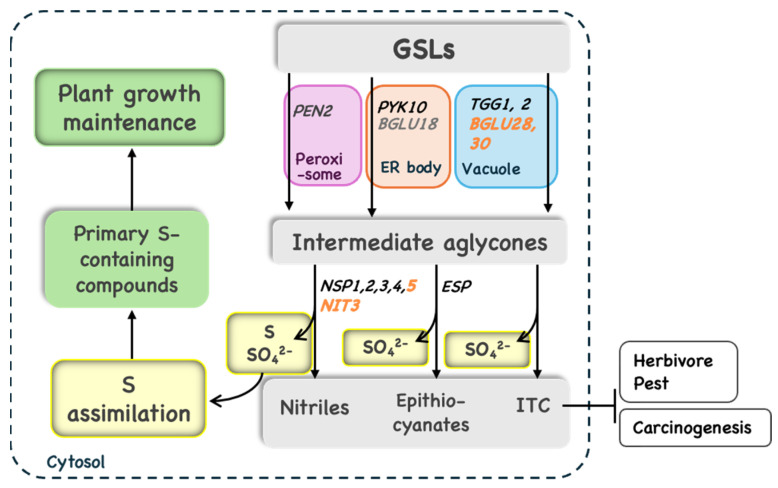
GSL catabolism contributes to various biotic and abiotic stresses and the reallocation of S. The enzyme names are shown in italics. GSL catabolic enzymes, BGLUs, exist and function in each subcellular compartment; PEN2 in peroxisome (pink), PYK10 and BGLU18 in ER body (orange), TGG1 and TGG2 in vacuole (blue). Subcellular localization of BGLU28 and BGLU30 are suggested to be vacuole. Enzyme names whose transcript levels increase under –S are shown in orange; GSL and GSL catabolites are shown on a grey background, S and SO_4_^2−^ on a yellow background, and terms related to primary S metabolism on a green background.

S deficiency also stimulates GSL catabolism, which starts with the cleavages of the thioglucosidic bond of GSL via the function of thioglucosidase (myrosinase), a kind of β-glucosidase (BGLU) (Figure 4). There are 47 types of BGLUs in *Arabidopsis*, 22 of which function as myrosinases [41]. These are classified into typical (BGLU34–BGLU39) and atypical (BGLU18-BGLU33) BGLUs according to the amino acid sequence of their catalytic centers. TGG1 (BGLU34) and TGG2 (BGLU35) have a wide range of substrate specificities and contribute to the generation of isothiocyanates (ITCs) caused by tissue damage [5,6]. iGSL-specific PEN2 (BGLU26) and PYK10 (BGLU23) contribute to pathogen resistance [7,8]. Based on the subcellular localization of PEN2 in peroxisomes and PYK10 in ER bodies, they are likely to be involved in intracellular GSL catabolism [41,42]. PYK10 functions as a determinant of the rhizosphere microbiome [43]. The contributions of other atypical BGLUs, such as BGLU18 localized in ER bodies and BGLU19 induced upon salinity stress, awaits further investigation [44,45]. A myrosinase-catalyzed reaction releases glucose from GSL, and the sulfate ion is spontaneously released from the unstable intermediate (Figure 1). The remaining aglycones are further metabolized to ITCs, epithiocyanates, or nitriles in the presence of a Specifier Protein [5,46,47,48].

Among BGLUs, two atypical BGLUs, BGLU28 and BGLU30, are induced in response to -S. The double, but not single, disruption lines of BGLU28 and BGLU30 accumulated significantly more GSLs than the wild-type under -S, accompanied by severe growth retardation [49,50]. In these plants, cysteine, GSH, and S levels in protein were also lower under -S than in wild-type plants [51]. These studies indicate that GSL catabolism by BGLU28 and BGLU30 is necessary for plant survival under -S by recycling S from GSLs to primary S metabolism, reaffirming the significance of GSLs in S storage (Figure 4).

S in ITC is also recycled for S assimilation, as demonstrated by the chase analysis of isotope-labeled GSL fed to plants [50]. Sulforaphane, a typical ITC produced after 4MSOB hydrolysis, is metabolized to raphanusamic acid (RA) after conjugation with GSH. RA contains an S atom derived from an ITC group and another S atom derived from GSH, from which two cysteine molecules are reproduced. In addition to ITC, nitriles and epithionitriles are produced via GSL catabolism, which typically occurs in disrupted tissues [51,52]. The nitrile pathway, which releases one more S moiety, can be beneficial for plant adaptation to -S, as suggested by the increased expression of nitrile pathway enzymes, *Nitrile Specifier Protein 5* (*NSP5*) and *Nitrilase3* (*NIT3*), under -S [32].

## 5. S-GSL Relationship Among Wild Perennial Brassicales

The close connection between S and GSL metabolism has been studied in different *Brassica* species, mainly focusing on cultivated *Brassica* crops [4,23,53]. Here, we review cases for wild perennial Brassicales, which generally grow in natural environments and develop unique adaptations to S availability [54].

Although less extensively explored than cultivated species, several studies have investigated the responses of S or S-related compounds in wild perennial plants. For example, when analyzing GSLs from two different habitats with low (2 to 4 ppm) and high levels (4 to 6 ppm) of both S and nitrate content in *Boechera stricta*, GSLs were found to be low regardless of the level of S and nitrate content, suggesting that the influence of one nutrient on the GSL profiles in *B. stricta* is dependent on the content of other nutrients [55]. Similar results were reported in *Armoracia rusticana,* a wild perennial plant native to Eastern Europe and Western Asia, which showed the highest percentage increase in GSLs of 150% by nitrogen (N) alone and 400% by N (100 kg ha^−1^) with S (45 kg ha^−1^) supply [56]. The plant is now widely cultivated for its roots because of the health benefits attributed by the presence of GSL and its bioactive catabolites such as AITC. A field study over two successive seasons in the short-lived perennial plant *Tropaeolum majus* indicated that S (100 kg ha^−1^) and N (120 kg ha^−1^) supply positively influenced benzyl GSL (gluco-tropaeolin) levels; however, the response of these elements varied between different plant parts and growth stages [57]. Therefore, the induction of GSL production in plants relies on both S and N levels in the soil. Furthermore, the application of selenium (a S analog) in a concentration of 2.5 to 10 mg per plant resulted in higher S and cysteine content in *Eruca sativa* (an annual plant), whereas their content decreased significantly in *Diplotaxis tenuifolia* (a perennial sp.) [58]. When analyzing the GSL content in both plants, the annual wild plant was found to accumulate higher amounts of GSLs, whereas the degradation of GSLs was observed in the perennial plant [58], which could have various ecological implications, including insect attack.

Owing to the positive impact of S on precursor molecules and GSLs, supplementation with various sulfate salts has become the key strategy to boost GSL levels in economically important *Brassica* vegetables and improve nutritional quality [59,60,61,62]. For instance, *B. rapa* seedlings accumulated more GSLs in response to a sulfate salt (50 mM Na_2_SO_4_) than chloride (50 mM NaCl and 50 mM KCl) ones [60], suggesting the potential to increase GSLs by modulating soil S levels. However, the effect of S supplementation on GSL levels varies among plant species, growth stages, organs, and types and concentrations of S used. For example, the GSL content in *B. rapa* was increased by Na_2_SO_4_ but not by K_2_SO_4_ [60]. Similarly, S fertilization increased the GSL content in the heads of some broccoli cultivars, whereas other cultivars did not respond or had even lower GSL content [23].

### Lepidium latifolium, a Special Case of Wild Perennial Brassicales

**Figure 5:**
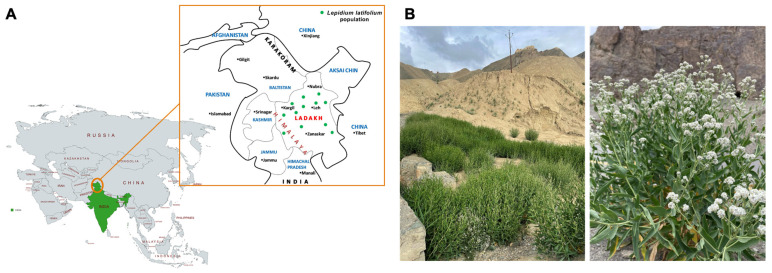
*Lepidium latifolium* growing in the trans-Himalayan region of Ladakh. (**A**) Location of Ladakh and the dotted green color representing distribution of *L. latifolium* throughout Ladakh Himalayas. (**B**) Images of *L. latifolium* growing at Leh in Ladakh (34°08.219′ N; 77°30.447′ E; 3164 m above sea level).

*L. latifolium* is a wild perennial plant commonly known as pepper weed, tall white top, pepperwort, peppergrass, or giant white weed (ironweed) (Figure 5). This plant is native to Southeastern Europe and is widely distributed in North America, Africa, and Asia [61,62]. In India, the plant has a wide native range across the trans-Himalayan region of Ladakh, where it grows at altitudes ranging from 2500 to 4500 m above sea level (Figure 5A). The trans-Himalayan region of Ladakh has harsh and unique ecosystem characteristics, with large temperature variations, sub-zero temperature, low annual precipitation (mostly in the form of snow), an intense radiation load, and a low partial pressure of gases (Figure 5B) [63]. In addition, the S level in the soil of Ladakh varied significantly among the different altitudinal sites [64]. More than eight different GSLs were identified, with 2-propenyl GSL (sinigrin) being predominant (>90%) [61,65]. Thus, the Himalayan ecotype of *L. latifolium* is one of the few wild perennial plants that have been extensively investigated for GSL metabolism and its role in biotic and abiotic stresses, including the trade-off between primary and secondary S metabolism, or vice versa. For example, S and GSL levels were analyzed at three native altitudinal sites with different soil S levels: Kargil (lower site, high S), Leh (middle site, moderate S), and Nyoma (higher site, low S). The S content in the leaves was higher at Kargil, corroborating the soil S content and indicating the efficient uptake of S from the soil. However, the GSL content appeared to be significantly higher at Leh, followed by Kargil [61], suggesting no direct correlation with soil S level. One reason for the lower GSL content in Kargil may be due to its hydrolysis by myrosinase in response to various environmental cues. The disintegration of GSL by myrosinase into various hydrolysis products that reallocate S into sulfate and cysteine in sprouts has also been demonstrated in this plant [66]. Unlike the majority of the Brassicaceae members, *L. latifolium* was found to produce a specific hydrolysis product from 2-propenyl GSL, CETP, suggesting the presence of a unique epithiospecifier protein—namely, Thiocyanate-Forming Protein (TFP) [66]. The cytoplasm-localized TFP protein belongs to the Kelch family and has a sequence and motif pattern similar to the ESP of *A. thaliana* [67]. The protein has a dual catalytic role, as it is responsible for forming epithionitrile, nitriles, and thiocyanate, depending upon the nature of GSL. For instance, it catalyzes the formation of epithionitrile, CETP, from 2-propenyl GSL, whereas it produces benzyl nitrile and benzyl thiocyanate from benzyl GSL in *Thlaspi arvensis* [68]. The different reallocation of S resources in mature plants may play a physiological role. To understand the connection between S and GSL metabolism, *L. latifolium* was supplemented with different concentrations (0.5 and 1.0 mM) of sulfate sources: magnesium sulfate (MgSO_4_), zinc sulfate (ZnSO_4_), and their combinations. These salts resulted in significant enhancements of GSL levels by up to 29 and 38% with MgSO_4_ and ZnSO_4_, respectively [65], suggesting that the mechanisms by which plants adjust their GSL profiles in response to S availability allow them to reallocate resources from growth to defense.

Due to the high mGSL content, particularly 2-propenyl GSL (sinigrin), and its therapeutic properties, *L. latifolium* is exploited for its nutraceutical potentials. Since its sprouts are rich sources of GSLs and their breakdown products, *L. latifolium* has been evaluated for its GSL profiles and other phytochemicals at different sprouts stages [66,69]. Unlike mature leaves, the sprouts of *L. latifolium* showed the predominant presence of another health-beneficial GSL, benzyl GSL (glucotropaeolin), suggesting that the third-week sprouts are a reservoir of phytochemicals and could be promoted as a functional food [69].

## 6. Rich GSL Diversity in Wild Perennial Brassicales

Extensive research has been conducted on GSL metabolism in the model plant *A. thaliana* and in economically important *Brassica* vegetables, including broccoli, cabbage, cauliflower, radish, and Brussels sprouts, to exploit the potential nutraceutical and pharmaceutical roles of GSL [70,71]. In addition, wild perennial Brassicales such as *Armoracia rusticana*, *L. latifolium*, *L. meyenii*, *Christolea crassifolia*, *Eruca sativa*, *Capparis spinosa*, *Diplotaxis tenuifolia*, *Matthiola fruiticulosa,* etc., have medicinal properties and form important parts of traditional diets. Among its therapeutic uses in traditional medicines, *L. latifolium* L. is used as a diuretic and anti-hypersensitive agent [61,72]. Similarly, the wild herb *E. sativa* has had a great culinary and medicinal history since Roman times, being used as a digestive and diuretic agent and as an aphrodisiac [73]. The therapeutic and medicinal properties of wild edible *Brassica* plants are attributed to the substantial amounts of GSLs and their hydrolysis products [62,69,74], which reduce the risk of multiple cancers in the rectum, lung, colon, and stomach [75,76,77]. Similarly, sulforaphane, the hydrolysis product of 4-(methylsulfinyl) butyl GSL (glucoraphanin), blocks the cell cycle and induces apoptosis, thereby preventing tumor growth [78,79]. These findings, combined with traditional knowledge of their medicinal properties, has promoted the use of wild *Brassica* crops to develop nutraceutical and pharmaceutical products [63,69,80]. Thus, researchers are focusing on modulating or boosting the desirable GSL levels in wild *Brassica* crops. S supplementation would likely become an important strategy to enhance the desirable GSLs in plants.

GSLs and their hydrolysis products are involved in several biological events too, such as stomatal conductance, flowering initiation, and heat stress mitigation [81,82,83,84], reflecting harsh environments such as drought, salinity, extreme temperatures, and high light. Indeed, each GSL molecule responds differently to abiotic stresses, and these effects can vary between plants, developmental stages, and plant organs [55,85]. In wild Brassicales, GSLs contribute to their ecological roles particularly in interactions with insects [75] and abiotic stress circumvention [86]. GSL diversity in the wild perennial plant *Cardamine hirsuta* is important for plant–insect, plant–environment, and plant–plant interactions [87]. The wild perennial plants *Draba borealis* and *C. fauriei* contain 11 and 3 GSL, respectively (Table 1) [88]. These GSLs are regulated by different light conditions, particularly by light quality, with the highest GSL content observed under blue and red light [89]. The ecological roles of GSLs in six wild species of the genus *Cardamine* were examined at an elevation of 2000 m. Abiotic factors associated with this elevation affect GSL biosynthesis, plant growth, and productivity. Moreover, *Pieris brassicae* induces GSLs in these species, indicating that increased GSL content confers resistance against insect herbivores [90]. *Arabis alpina* is a short-lived perennial species widely distributed in alpine environments across Europe, North and East Africa, Central and Eastern Asia, and North America [91]. Twenty-one different GSLs were identified in the sixteen field-surveyed populations of *A. alpina,* with three, 3-butenyl GSL (gluconapin), 2-hydroxy-3-butenyl GSL (progoitrin), and 9-(methylsulfinyl) nonyl GSL (glucoarabin), constituting more than 70% of the total (Table 1). Although the total GSL levels were uncorrelated with the increasing altitudinal gradient, despite a 2.9-fold difference in the total GSL content across populations, individual GSL levels were affected by elevation and population size. Furthermore, leaf herbivory results in a stronger induction of GSLs in high-altitude plants than in intermediate- or low-altitude plants [92]. Similarly, *Aethionema saxatile* upon herbivory by *Plutella xylostella* led to iGSL accumulation not only in the leaves but also in the diaspores, which required optimal defense after seed dispersal [93]. *L. meyenii* relocates and remobilizes benzenic GSL to cope with strong UV radiation at high altitudes [94].

Studies on GSL metabolism in wild perennial Brassicales plants suggest that these plants are useful for studying adaptations to the expected rise in both biotic and abiotic (temperature) pressures at high altitudes due to climate change. Considering the diverse array of GSLs among wild perennial Brassicales (Table 1), the exploitation of different wild perennial species for their GSLs could result in the identification of novel GSLs with more efficient nutraceutical, pharmaceutical, ecological, and physiological roles than known ones. A detailed study of GSL function and the regulatory mechanisms underlying their accumulation in wild perennial species would help to identify the critical factors associated with S and GSL metabolism.

## 7. Conclusions

GSL metabolism in plants is complex and is significantly influenced by environmental S availability. The involvement of various important factors, such as SLIM1, SDIs, BGLU28, and BGLU30, in the trade-off between GSL and primary S metabolism mitigates the ability of plants to withstand S-deficient conditions. Similarly, wild perennial Brassicales exhibit unique mechanisms for adjusting their GSL profiles, enabling them to balance defense and growth under varying S conditions. These plants, which are known for their medicinal and ecological importance, offer valuable potential for the development of nutraceutical and pharmaceutical products because of their large diversity of GSLs and the hydrolysis products. Further research on S and GSL metabolism in wild perennial Brassicales, along with their ecological roles in biotic and abiotic stress adaptation, could reveal novel GSL and the functions with enhanced ecological implications. Understanding the regulatory mechanisms underlying S and GSL metabolism in model and wild Brassicales would help to identify key factors and other metabolic processes associated with them.

## Figures and Tables

**Table 1:** List of wild perennial Brassicales and the respective glucosinolates (GSLs), depicting the rich diversity of GSLs. Names of plants with the highest GSL content and the GSLs with the highest accumulation in each plant are shown with bold characters.

Plant Name	Glucosinolates						Refs
	Aliphatic		Indolic		Aromatic/Benzenic		
	Chemical Name	Common Name	Chemical Name	Common Name	Chemical Name	Common Name	
*Diplotaxis* *viminea*	5-(methylsulfinyl)pentyl GSL 1-methylpropyl GSL 4-(methylthio)butyl GSL **4-(methylsulfonyl)butyl GSL** 7-(methylsulfinyl)heptyl GSL 3-(methylthio)propyl GSL **3-methylbutyl GSL** **ethyl GSL** 3-butenyl GSL isopropyl GSL 4-(methylsulfinyl)butyl GSL 4-(methylsulfinyl)-3-butenyl GSL **4-(mercaptobutyl) GSL** 2-hydroxy-3-butenyl GSL, 2-propenyl GSL **4-(glucopyranosyldithio)butyl GSL** **Dihydrogluconapin** 3-hydroxy-5-(methylsulfinyl)pentyl GSL	Glucoalyssin Glucocochlearin Glucoerucin **Glucoerysihienin** Glucoibarin Glucoiberverin **Glucojiaputin** **Glucolepidiin** Gluconapin Glucoputranjivin Glucoraphanin Glucoraphenin **Glucosativin** Progoitrin Sinigrin **Diglucothiobeinin** **Dihydrogluconapin** -	indol-3-ylmethyl GSL 4-hydroxyindol-3-ylmethyl GSL 4-methoxyindol-3-ylmethyl GSL 1-methoxyindol-3-ylmethyl GSL	Glucobrassicin 4-Hydroxyglucobrassicin 4-Methoxyglucobrassicin Neoglucobrassicin	2-phenylethyl GSL 4-hydroxybenzyl GSL benzyl GSL **4-(rhamnopyranosyloxy)bezyl GSL** **2-(benzoyloxy)ethyl GSL**	Gluconasturtin Sinalbin Glucotropaeolin **Glucoamoracin** -	[80]
*Draba* *borealis*	3-(methylsulfinyl)propyl GSL 4-(methylsulfinyl)butyl GSL 5-(methylsulfinyl)pentyl GSL 1-methylpropyl GSL 3-(methylthio)propyl GSL 6-(methylsulfinyl)hexyl GSL **5-(methylthio)pentyl GSL** **6-(methylthio)hexyl GSL** 4-(methylthio)butyl GSL 4-methoxy-3-indolylmethyl GSL **9-(methylsulfinyl)nonyl GSL**	Glucoiberin Glucoraphanin Glucoalyssin Glucocochlearin Glucoibervirin Glucohesperin **Glucoberteroin** **Glucolesquerellin** Glucoerucin Glucohirsutin **Glucoarabin**					[88]
*Cardamine* sp.	2-hydroxy-3-butenyl GSL 3-butenyl GSL 7-(methylsulfinyl)heptyl GSL 4-pentenyl GSL	Progoitrin Gluconapin Glucoibarin Glucobrassicanapin	indol-3-ylmethyl GSL	Glucobrassicin	4-hydroxybenzyl GSL benzyl GSL	Sinalbin Glucotropaeolin	[89,90]
*Arabis* *alpina*	2-hydroxy-3-butenyl GSL 5-(methylsulfinyl)pentyl GSL 4-(methylsulfinyl)butyl GSL 3-butenyl GSL 4-(methylthio)butyl GSL **9-(methylsulfinyl)nonyl GSL** 4-pentenyl GSL 4-methoxy-3-indolylmethyl GSL **5-(methylthio)pentyl GSL** **10-(methylsulfinyl)decyl GSL** **7-(methylthio)heptyl GSL** 8-(methylthio)octyl GSL **9-(methylthio)nonyl GSL** **9-(methylsulfonyl)nonyl GSL**	Progoitrin Glucoalyssin Glucoraphanin Gluconapin Glucoerucin **Glucoarabin** Glucobrassicanapin Glucohirsutin **Glucoberteroin** **Glucocamelinin** - - - -	4-methoxyindol-3-ylmethyl GSL	4-Methoxyglucobrassicin			[92]
*Lepidium* *latifolium*	3-(methylsulfinyl)propyl GSL 3-(methylsulfonyl)propyl GSL 2-propenyl GSL 5-(methylsulfinyl)pentyl GSL 3-butenyl GSL 1-methylpropyl GSL 3-(methylsulfonyl)propyl GSL	Glucoiberin Glucocherolin Sinigrin Glucoalyssin Gluconapin Glucocochlearin Glucocheirolin	4-methoxyindol-3-ylmethyl GSL	4-Methoxyglucobrassicin	benzyl GSL 2-phenylethyl GSL	Glucotropaeolin Gluconasturtiin	[61,69,70,95]
*Degenia* *velebitica*	5-(methylthio)pentyl GSL 4-pentenyl GSL 5- (methylsulfinyl)pentyl GSL 4-(methylthio)butyl GSL	Glucoberteroin Glucobrassicanapin Glucoalyssin Glucoerucin			**4-methoxybenzyl GSL**	**Glucoaubrietin**	[96]
*Diplotaxis* *tenuifolia*	**4-(glucopyranosyldisulfanyl)butyl GSL** 5-(methylsulfinyl)pentyl GSL 4-(methylthio)butyl GSL 7-(methylsulfinyl)heptyl GSL 3-(methylthio)propyl GSL **ethyl GSL** 3-butenyl GSL 4-pentenyl GSL **isopropyl GSL** 4-(methylsulfinyl)butyl GSL 4-(methylsulfinyl)-3-butenyl GSL **4-(mercaptobutyl) GSL** 2-hydroxy-3-butenyl GSL 2-propenyl GSL	**Diglucothiobeinin** Glucoalyssin Glucoerucin Glucoibarin Glucoiberverin **Glucolepidiin** Gluconapin Glucobrassicanapin **Glucoputranjivin** Glucoraphanin Glucoraphenin **Glucosativin** Epigoitrin Sinigrin	indol-3-ylmethyl GSL 1-methoxyindol-3-ylmethyl GSL	Glucobrassicin Neoglucobrassicin	2-phenylethyl GSL benzyl GSL 4-hydroxybenzyl GSL -	Gluconasturtin Glucotropaeolin Sinalbin **Glucoarmoracialafolicin**	[97]
*Hesperis* *matronalis*	3-(methylthio)propyl GSL 4-(methythio)butyl GSL **5-(methylthio)pentyl GSL** **6-(methylthio)hexyl GSL** 5-(methylsulfinyl)pentyl GSL **6-(methylsulfinyl)hexyl GSL** 1-methylpropyl GSL 3-butenyl GSL 2-hydroxy-3-butenyl GSL	Glucoibervirin Glucoerucin **Glucoberteroin** **Glucolesqurellin** Glucoalyssin **Glucohesperin** Glucocochlearin Gluconapin Epigoitrin			benzyl GSL 4-hydroxybenzyl GSL **3,4-dihydroxybenzyl GSL**	Glucotropaeolin Sinalbin **Glucomatronalin**	[98]
*Isatis* *tinctoria*	2-hydroxy-3-butenyl GSL 2-hydroxy-3-butenyl GSL 3-butenyl GSL	Epigoitrin Progoitrin Gluconapin	**Sulfoindol-3-ylmethyl GSL** 4-hydroxyindol-3-ylmethyl GSL 4-methoxyindol-3-ylmethyl GSL 1-methoxyindol-3-ylmethyl GSL indol-3-ylmethyl GSL	**Sulfoglucobrassicin** 4-Hydroxyglucobrassicin 4-Methoxyglucobrassicin Neoglucobrassicin Glucobrassicin	-	**Glucoisatisin**	[99]
*Lepidium* *draba*	3-butenyl GSL **ethyl GSL** 4-(methythio)butyl GSL **iso-butyl GSL** 4-(methylsulfinyl)butyl GSL 3-(methylthio)propyl GSL 1-methylpropyl GSL 4-pentenyl GSL	Gluconapin **Glucolepidiin** Glucoerucin - Glucoraphanin Glucoibervirin Glucocochlearin Glucobrassicanapin			benzyl GSL	Glucotropaeolin	[100]
*Lepidium* *meyenii*	5-(methylsulfinyl)pentyl GSL 4-pentenyl GSL	Glucoalyssin Glucobrassicanapin	4-methoxyindol-3-ylmethyl GSL indol-3-ylmethyl GSL	4-Methoxyglucobrassicin Glucobrassicin	benzyl GSL **3-methoxybenzyl GSL** 2-hydroxybenzyl GSL **3-Hydorxybenzyl GSL** **4-methoxybenzyl GSL**	Glucotropaeolin **Glucolimnanthin** Glucosinalbin **Glucolepigramin** **Glucoaubrietin**	[101]
*Lunaria* *annua*	**isopropyl GSL** **6-(methylsulfinyl)hexyl GSL** 5-(methysulfinyl)pentyl GSL **2-hydroxy-4-pentenyl GSL** 1-methylpropyl GSL	**Glucoputranjivin** **Glucohesperin** Glucoalyssin **Gluconapoleiferin** Glucocochlearin	1-methoxyindol-3-ylmethyl GSL	Neoglucobrassicin	2-phenylethyl GSL	Gluconasturtiin	[102]
*Nasturtium* *officinale*	3-(methylsulfinyl)propyl GSL 4-(methylsulfinyl)butyl GSL **10-(methylsulfinyl)decyl GSL** 2-hydroxy-4-pentenyl GSL **9-(methylsulfinyl)nonyl GSL** 4-pentenyl GSL 3-(methylsulfonyl)propyl GSL **ethyl GSL** **5-(methylthio)pentyl GSL** 4-(methylthio)butyl GSL 2-propenyl GSL 3-butenyl GSL 2-hydroxy-3-butenyl GSL 7-(methylsulfinyl)heptyl GSL 8-(methylsulfinyl)octyl GSL	Glucoiberin Glucoraphanine **Glucocamelinin** Homoglucocamelinin **Glucoarabin** Glucobrassicanapin Glucocherolin **Glucolepidiin** **Glucoberteroin** Glucoerucin Sinigrin Gluconapin Progoitrin Glucosiberin Glucohirsutin	4-hydroxyindol-3-ylmethyl GSL 4-methoxyindol-3-ylmethyl GSL indol-3-ylmethyl GSL 1-methoxyindol-3-ylmethyl GSL	4-Hydroxyglucobrassicin, 4-Methoxyglucobrassicin Glucobrassicin Neoglucobrassicin	benzyl GSL 2-hydroxybenzyl GSL 2-phenylethyl **4-rhamnosyloxybenzyl GSL** **3-methoxybenzyl GSL**	Glucotropaeolin Sinalbin Gluconasturtiin **Glucomoringin** **Glucolimnanthin**	[103,104]
*Pringlea antiscorbutica*	4-(methylsulfinyl)butyl GSL 2-propenyl GSL 4-(methylthio)butyl GSL n-butyl GSL 3-butenyl GSL	Glucoraphanin Sinigrin Glucoerucin - Gluconapin			benzyl GSL	Glucotropaeolin	[105]
*Rorippa* *austriaca*	7-(methylsulfinyl)heptyl GSL **6-(methylsulfinyl)hexyl GSL** 8-(methylsulfinyl)octyl GSL **5-(methylthio)pentyl GSL**	Glucoibarin **Glucochesperin** Glucohirsuitin **Glucoberteroin**	indol-3-ylmethyl GSL 4-methoxyindol-3-ylmethyl GSL	Glucobrassicin 4-Methoxyglucobrassicin			[106]

## Abbreviations

The following abbreviations are used in this manuscript:

GSL	Glucosinolate
ITC	Isothiocyanate
mGSL	Aliphatic glucosinolate
iGSL	Indolic glucosinolate
I3M	indol-3-ylmethyl GSL
1MOI3M	1-methoxy I3M
4MOI3M	4-methoxy I3M
MSOX	Methylsulfinylalkyl
MTX	Methylthioalkyl
3MSOP	3-methylsulfinylpropyl
4MSOB	4-methylsulfinylbutyl
7MSOH	7-methylsulfinylheptyl
8MSOO	8-methylsulfinyloctyl
4MTB	4-Methylthiobutyl
7MTH	7-methylthioheptyl
8MTO	8-methylthiooctyl
GTR	Glucosinolate transporter
UMAMIT	Usually multiple acids move in out transporter
BCAT	Branched-chain amino acid aminotransferase
MAM	Methylthioalkylmalate synthase
IPMI	Isopropylmalate isomerase
IPMDH	Isopropylmalate dehydrogenase
CYP	Cytochrome P450
GSH	Glutathione
GST	GSH-S-transferase
GGP	γ-glutamyl peptidase
SUR	S-alkyl-thiohydroxy acid lyase
SOT	Sulfo-transferase
PAPS	3′-phosphoadenosine 5′-phosphosulfate
FMO-GSOX	Flavin-containing monooxygenase
SLIM	Sulfur limitation
EIL	Ethylene-Insensitive3-Like
UGT	Glucosyltransferases of the UGT74 family
AOP	2-oxoglutarate-dependent dioxygenase
GS-OH	2-oxo acid-dependent dioxygenase
LSU	Response to low sulfur
SDI	Sulfur deficiency induced
TFP	Thiocyanate-Forming Protein
TPR	Tetratricopeptide repeat
BGLU	β-glucosidase
TGG	Thioglucoside glucohydrolase
PEN	Penetration
NSP	Nitrile specifier protein
NIT	Nitrilase
ESP	Epithiospecifier protein
RA	Raphanusamic acid
AITC	Allyl-isothiocyanate
CETP	1-Cyano-2,3-epithiopropane
